# Role of Annexin A1 in NLRP3 Inflammasome Activation in Murine Neutrophils

**DOI:** 10.3390/cells10010121

**Published:** 2021-01-11

**Authors:** José Marcos Sanches, Rebeca D. Correia-Silva, Gustavo H. B. Duarte, Anna Maria A. P. Fernandes, Salvador Sánchez-Vinces, Patrícia O. Carvalho, Sonia M. Oliani, Karina R. Bortoluci, Vanessa Moreira, Cristiane D. Gil

**Affiliations:** 1 Programa de Pós-Graduação em Biologia Estrutural e Funcional, Universidade Federal de São Paulo, São Paulo 04023-900, Brazil; ms.marcossanches@gmail.com (J.M.S.); beca97c@gmail.com (R.D.C.-S.); sonia.oliani@unesp.br (S.M.O.); 2 Instituto de Química, Universidade Estadual de Campinas, Campinas 13083-862, São Paulo, Brazil; gustavo_duarte95@hotmail.com; 3 Laboratório de Pesquisa Multidisciplinar, Universidade São Francisco, Bragança Paulista 12916-900, São Paulo, Brazil; annamabr@yahoo.com.br (A.M.A.P.F.); ssanchez@ime.usp.br (S.S.-V.); patricia.carvalho@usf.edu.br (P.O.C.); 4 Programa de Pós-Graduação em Biociências, Universidade Estadual Paulista (UNESP), Instituto de Biociências Letras e Ciências Exatas, São José do Rio Preto 15054-000, São Paulo, Brazil; 5 Departamento de Ciências Biológicas e Centro de Terapia Celular e Molecular, Universidade Federal de São Paulo, São Paulo 04044-010, Brazil; karina.bortolucci@unifesp.br; 6 Departamento de Farmacologia, Universidade Federal de São Paulo, São Paulo 04044-020, Brazil; vmoreira@unifesp.br

**Keywords:** Ac_2-26_, ATP, inflammation, mass spectrometry, nigericin, lipidomics

## Abstract

This study evaluated the role of endogenous and exogenous annexin A1 (AnxA1) in the activation of the NLRP3 inflammasome in isolated peritoneal neutrophils. C57BL/6 wild-type (WT) and AnxA1 knockout mice (AnxA1^-/-^) received 0.3% carrageenan intraperitoneally and, after 3 h, the peritoneal exudate was collected. WT and AnxA1^-/-^ neutrophils were then stimulated with lipopolysaccharide, followed by the NLRP3 agonists nigericin or ATP. To determine the exogenous effect of AnxA1, the neutrophils were pretreated with the AnxA1-derived peptide Ac_2-26_ followed by the NLRP3 agonists. Ac_2-26_ administration reduced NLRP3-derived IL-1β production by WT neutrophils after nigericin and ATP stimulation. However, IL-1β release was impaired in AnxA1^-/-^ neutrophils stimulated by both agonists, and there was no further impairment in IL-1β release with Ac_2-26_ treatment before stimulation. Despite this, ATP- and nigericin-stimulated AnxA1^-/-^ neutrophils had increased levels of cleaved caspase-1. The lipidomics of supernatants from nigericin-stimulated WT and AnxA1^-/-^ neutrophils showed potential lipid biomarkers of cell stress and activation, including specific sphingolipids and glycerophospholipids. AnxA1 peptidomimetic treatment also increased the concentration of phosphatidylserines and oxidized phosphocholines, which are lipid biomarkers related to the inflammatory resolution pathway. Together, our results indicate that exogenous AnxA1 negatively regulates NLRP3-derived IL-1β production by neutrophils, while endogenous AnxA1 is required for the activation of the NLRP3 machinery.

## 1. Introduction

NLRP3 (NOD-like receptor pyrin domain-containing protein 3, cryopyrin, or NALP3) is the most studied member of the intracellular pattern recognition NOD (nuclear-binding and oligomerization domain)-like receptors [1,2]. The NLRP3 inflammasome is activated by a broad range of pathogen- and damage-associated molecular patterns, such as adenosine triphosphate (ATP), uric acid crystals and β-amyloid plaques, as well as environmental irritants [2]. Inflammasome activation, which consists of a sensor (NLRP3), an adaptor (apoptosis-associated spec-like protein, ASC; also known as PYCARD), and an effector (caspase 1), culminates in the release of mature IL-1β and IL-18 forms through membrane pore formation by gasdermin D cleavage [1,2]. The dysregulation of the NLRP3 inflammasome pathway is associated with the development of many human diseases, such as atherosclerosis, metabolic syndromes, age-related macular degeneration, Alzheimer’s disease, and gout [3,4]. Therefore, investigations of novel signaling components that regulate inflammasome activation are crucial to prevent or treat human inflammatory diseases.

Annexin A1 (AnxA1) is a glucocorticoid-regulated protein that controls molecular and cellular steps involved in the inflammatory response, such as the inhibition of phospholipase A2 and eicosanoid synthesis, leukocyte transmigration, pro-inflammatory cytokine production, and clearance of apoptotic neutrophils by macrophages [5]. In addition, AnxA1 N-terminal-derived peptides mimic the pharmacological property of the whole protein, especially its anti-inflammatory activity, by binding to a specific class of G-protein coupled receptor, the formyl peptide receptors (Fprs) [5,6]. Studies by our group and others have shown that the administration of these peptidomimetics, especially Ac_2-26_, can downregulate inflammatory responses, including the release of IL-1β, in several experimental rodent models including myocardial and intestinal ischemia reperfusion [7,8,9], endotoxin-induced uveitis [10], acute gouty inflammation [11], and pilocarpine-induced status epilepticus [12].

Recently, the role of AnxA1 in NLRP3 activation was demonstrated in murine macrophages [13,14]. Macrophages from AnxA1 null (AnxA1^-/-^) mice showed increased levels of NLRP3 compared to wild-type cells and produced a marked increase in IL-1β levels after stimulation with the potassium ionophore nigericin [13]. Additionally, the lipidomic analysis of WT and AnxA1^-/-^ cell supernatants revealed a distinct set of lipid profiles under NLRP3 stimulation, suggesting that the lack of AnxA1 favors lipopolysaccharide (LPS) “over-priming” and the release of lipid mediators (e.g., ceramides) that induce a prominent NLRP3 activation under nigericin stimulation.

Neutrophils are essential immune cells with functions mediated by cell surface receptors, including G-protein-coupled receptors, adhesion molecules, Fc-receptors, Toll-like receptors, and cytokine receptors, which trigger diverse cell signaling pathways [15]. The activation of these receptors, including by many lipid mediators, can lead to a range of neutrophil activation processes, including the exocytosis of granule contents, reactive oxygen species (ROS) production, phagocytosis, release of neutrophil extracellular traps (NETs), migration, and cytokine/chemokine release [15]. The NLRP3 inflammasome pathway is also operational in neutrophils, which are the major source of IL-1β in bone marrow in response to classical NLRP3 activators (ATP and nigericin) [16]. However, the pathways involved in the induction of NLRP3 activation and inflammasome formation in neutrophils remain to be elucidated.

Building upon these observations that AnxA1^-/-^ neutrophils present enhanced endothelial transmigration and increased responsiveness to inflammatory stimuli [17], this study evaluates the role of endogenous and exogenous AnxA1 on NLRP3 activation in neutrophils and also adds a new perspective on their lipid metabolism.

## 2. Materials and Methods

### 2.1. Animals

Male C57BL/6 wild-type (WT) and AnxA1^-/-^ mice 7–8 weeks of age with a weight of 20–25 g were kept in cages (four mice per cage) in a temperature-controlled environment (22–25 °C) on a 12 h light-dark cycle and received water and food *ad libitum*. All animal procedures were approved by the Ethics Committee in Animal Experimentation of the Federal University of São Paulo-UNIFESP (CEUA agreement number: N° 6493130318) and by the Internal Biosafety Commission.

### 2.2. Cell Culture and Treatments

LPS, nigericin, and ATP were obtained from InvivoGen (San Diego, CA, USA). LPS and ATP were reconstituted in endotoxin-free water and nigericin in 100% ethanol. The stock solution was diluted in endotoxin-free water to prepare an intermediate concentration solution and stored at −20 °C. The AnxA1-derived peptide Ac_2-26_ (Ac-AMVSEFLKQAWFIENEEQEYVQTVK; Invitrogen, São Paulo, Brazil) was reconstituted in Opti-MEM (Thermo Fisher Scientific, Waltham, MA, USA) and stored at −20 °C.

WT and AnxA1^-/-^ peritoneal neutrophils were obtained by the intraperitoneal injection of a 0.3% carrageenan solution in sterile PBS, and after three hours, cells were collected by peritoneal wash. Differential cell counts were made on Diff-Quick-stained cell smears prepared by cytocentrifugation. The neutrophil population obtained was more than 84–90% pure and at least 90% viable, as examined by trypan blue exclusion. Additionally, neutrophil morphology was confirmed by ultrastructural analysis using transmission electron microscopy. Peritoneal cells were cultured at 37 °C with ≤ 5% CO_2_ atmosphere at 1 × 10^6^ cells in Opti-MEM (Thermo Fisher Scientific, Waltham, MA, USA) in 1.5 mL conical microtubes. The experiments were performed in triplicate or quadruplicate in independent times, in 1.5 mL conical microtubes. WT and AnxA1^-/-^ cells were primed with LPS (500 ng/mL for 3 h) followed by stimulation with nigericin (10 μM for 1 h) or ATP (5 mM, 30 min) to activate the NLRP3 inflammasome. Additionally, a set of primed WT and AnxA1^-/-^ neutrophils were treated with Ac_2-26_ (1.6 μM) for 15 min before NLRP3 activation by nigericin or ATP.

### 2.3. Cytotoxicity Assay by Lactate Dehydrogenase Release

Lactate dehydrogenase (LDH) release was determined using the Pierce LDH Cytotoxicity Assay Kit (Thermo Fisher Scientific, Waltham, MA, USA), according to the manufacturer’s instructions. 1 × 10^4^ WT and AnxA1^-/-^ cells were challenged as described previously [18] and the supernatants were collected to perform the LDH assay. LDH positive controls were obtained by lysing WT and AnxA1^-/-^ cells with a solution containing 1% Triton X100 in Opti-MEM (Thermo Fisher Scientific, Waltham, MA, USA). Absorbance (at 490 nm and 680 nm) was measured using a plate reader (800™ TS Absorbance Reader, Biotek, Winooski, VT, USA). All experiments were carried out in triplicate, and the data were expressed as the mean ± standard error of the mean (SEM) optical density.

### 2.4. IL-1β and Caspase 1 Levels

IL-1β and caspase 1 release were tested in culture supernatants by enzyme-linked immunosorbent assay (ELISA) using commercially available immunoassay kits (IL-1β: BioLegend, San Diego, CA, USA; caspase 1: XpressBio, Frederick, MD, USA) following the manufacturer’s instructions. All experiments were conducted in duplicate, and the data were expressed as the mean ± SEM of protein (pg/mL).

### 2.5. Western Blot Analysis

After treatment, WT and AnxA1^-/-^ cells were washed three times with sterile PBS, and 50 µL of lysis buffer was added in each tube for neutrophil lysis and protein extraction. Cell extracts were loaded onto a 12% sodium dodecyl sulfate-polyacrylamide gel for electrophoresis together with appropriate molecular weight markers (Bio-Rad Life Science, Hercules, CA, USA) and transferred to ECL Hybond nitrocellulose membranes. Reversible protein staining of the membranes with 0.1% Ponceau-S in 5% acetic acid (Santa Cruz Biotechnology, Paso Robles, CA, USA) was used to verify protein transfer. The membranes were incubated for 30 min in 5% milk in Tris-buffered saline (TBS) before incubation with antibodies. Primary antibodies used were as follows: rabbit polyclonal anti-AnxA1 (Invitrogen-Thermo Fisher Scientific, Waltham, MA, USA; 1:1000), goat polyclonal anti-IL-1β (R&E Systems, Minneapolis, MN, USA; 1:500), mouse monoclonal anti-caspase-1 (Santa Cruz Biotechnology, Paso Robles, CA, USA; 1:200) and polyclonal rabbit anti-β-actin (Cell Signaling Technology, Danvers, MA, USA; 1:1000), all diluted in TBS. Then, the membranes were incubated with the appropriate peroxidase-conjugated secondary antibodies (1:2500; Millipore Corporation, Burlington, CA, USA). Finally, membranes were washed for 15 min with TBS, and immunoreactive proteins were detected (Clarity™ Western ECL Substrate; Bio-Rad, Hercules, CA, USA) using a GeneGnome5 chemiluminescence detection system (SynGene, Cambridge, UK). Proteins were then imaged and quantified using ImageJ 1.53e software) to determine the relative expression of the indicated proteins (arbitrary units, a.u.).

### 2.6. Ultrastructural Immunocytochemical Analysis

WT and AnxA1^-/-^ nigericin-stimulated neutrophils were processed for transmission electron microscopy analysis as previously described [13]. To detect NLRP3, ultrathin sections (~90 nm) from neutrophils were submitted for immunocytochemistry, using a rabbit polyclonal antibody anti-NLRP3 1:200 (Cusabio, Houston, TX, USA) followed by a goat anti-rabbit IgG antibody (1:50) conjugated to 15 nm colloidal gold (British Biocell, Cardiff, UK). Ultrathin sections were stained with uranyl acetate and lead citrate and examined using a ZEISS EM900 electron microscope (Carl Zeiss, Oberkochen, Germany).

### 2.7. Untargeted Mass Spectrometry Lipidomic Analysis

After treatment, WT and AnxA1^-/-^ cell supernatants (1 × 10^6^ cells/well) were collected and stored at −80 °C until sample processing. For lipid extraction, samples were randomized and resuspended in 380 µL of 1:2 CHCl_3_:MeOH solution (Sigma Aldrich, Basel, Switzerland), followed by the addition of 125 µL CHCl_3_ and 125 µL deionized water. The solution was then stirred for 5 min, followed by centrifugation at 13,000 rpm for 5 min. the derived organic fractions with lipids were collected from the bottom layer of the tubes and transferred to 2 mL glass tubes. These fractions were dried in a concentrator SpeedVac Savant SPD131DDA (Thermo Scientific, Waltham, MA, USA) for 30 min at 30 °C and kept frozen at −80 °C.

Dried lipid extracts were reconstituted in 1 mL of a 50 µg/mL solution of P-fluoro-DL-phenylalanine (PFFA) diluted in isopropanol/acetonitrile/water (2:1:1, *v/v/v*) before liquid chromatography–mass spectrometry (LC-MS) analysis. PFFA, used as internal standard, formic acid, and ammonium acetate were from Sigma-Aldrich (Saint Louis, MO, USA). Water was purified on a Milli-Q system from Millipore (Medford, MA, USA). HPLC-grade acetonitrile (ACN) and isopropanol were from Honeywell (Morristown, NJ, USA). Data were acquired using an ACQUITY FTN liquid chromatograph coupled to a XEVO-G2XSQTOF mass spectrometer (Waters, Milford, MA, USA) using MassLynx 4.1 software as previously described [19]. Briefly, a Supelcosil LC-18 (2.1 × 100 mm^2^, 5.0 µm, Supelco) column was used. The mobile phase consisted of: (A) 10 mM of ammonium acetate with 0.1% formic acid in acetonitrile/water (60:40, *v/v*) and (B) 10 mM ammonium acetate with 0.1% formic acid in isopropanol/water (90:10, *v*/*v*) at a flow rate of 0.40 mL/min with a linear gradient (in % B): 0–2.0 min: 40–43%; 2.0–2.1 min: 43–50%; 2.1–12.0 min: 50–54%; 12.0–12.1 min: 54–70%; 12.1–18 min: 70–99%; 18–18.1 min: decrease to 40% (with a further 1.8 min for column re-equilibration), resulting in a 20 min analysis. The injection volume was 1 µl. For the electrospray ionization source, the parameters were set as follows: positive mode with a capillary voltage of 3.5 kV, sampling cone of 40,000, source temperature of 140 °C, desolvation temperature of 550 °C, cone gas flow of 10 L/h, and desolvation gas flow of 900 L/h; negative mode with capillary voltage of 2.5 V, sampling cone of 40,000, source temperature of 140 °C, desolvation temperature of 550 °C, cone gas flow of 50 L/h, and desolvation gas flow of 900 L/h. The acquisition scan range was from 50 to 1700 Da and the data were acquired using a high energy mass spectrometry (MS^E^) approach. Leucine encephalin (molecular weight = 555.62; 200 pg/μL in 1:1 ACN:H_2_O) was used as a lock mass for accurate mass measurements, and a 0.5 mM sodium formate solution was used for instrument calibration. The samples were randomly analyzed to observe the biological variation and minimize instrumental bias. To monitor the stability of the system, quality control (QC) samples were inserted 15 times before the batch and inserted after eight injections [20].

Raw data were processed with Progenesis QI 2.0 software (Nonlinear Dynamics, Newcastle, UK) for peak detection, alignment, integration, deconvolution, data filtering, ion annotation, and MS^E^ based putative identification of compounds. Lipid Maps databases (http://www.lipidmaps.org/), LipidBlast (https://fiehnlab.ucdavis.edu/projects/LipidBlast) and Human Metabolome Database (http://www.hmdb.ca/metabolites) were used for this identification with the search parameters of precursor mass error ≤ 5 ppm and fragment tolerance ≤ 10 ppm. Fragmentation score, mass accuracy, and isotope similarity were considered for the identification of the molecules.

### 2.8. Statistical Analysis

The data were analyzed using GraphPad Prism 5.0 software. The results were confirmed to follow a normal distribution using the Kolmogorov–Smirnov test of normality with Dallal–Wilkinson–Lillie for corrected *p*-value. The data that passed the normality assumption were analyzed using analysis of variance (ANOVA) with Bonferroni post hoc test. Data failing the normality assumption were analyzed using the non-parametric Kruskal–Wallis test followed by Dunn’s post-test, and differences were considered statistically significant at *p* < 0.05.

Before the statistical analysis of lipidomics data, the peak area of each feature was normalized by the internal standard. Afterward, statistical analysis was performed using the MetaboAnalyst 4.0 web platform (McGill University, Montreal, QC, Canada). Data was raw-wised normalized and range-scaled before performing statistical analysis. Unpaired analysis of variance (ANOVA) was used as a univariate statistical analysis and Fisher’s least significant difference method correction was applied. Only features (i.e., an ion with a unique m/z and retention time) that fulfilled the criteria of *p*-value false discovery rate (FDR) adjusted < 0.05 and FDR < 0.05 were considered as significant. FDR adjusted *p*-value in addition to hierarchical analysis was used to construct the heat map.

## 3. Results

### 3.1. NLRP3 Inflammasome-Derived IL-1β Production is Impaired in AnxA1^-/-^ Neutrophils

**Figure 1:**
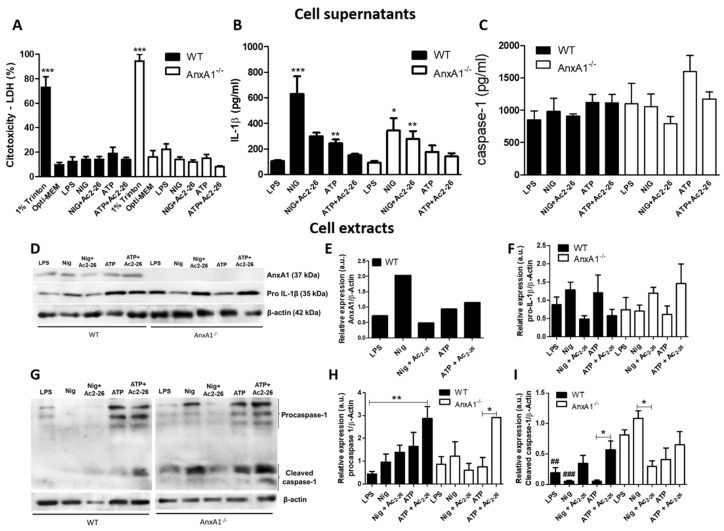
NLRP3 inflammasome activation in wild-type (WT) and AnxA1^-/-^ neutrophils. WT and AnxA1^-/-^ neutrophils were primed with lipopolysaccharide (LPS) and then stimulated with ATP, ATP with Ac_2-26_ pre-treatment (A + Ac_2-26_), nigericin (Nig) and nigericin with Ac_2-26_ pre-treatment (Nig + Ac_2-26_). Cell supernatants (**A**–**C**). Cell Extracts (**D**–**I**). (**A**) Lactate dehydrogenase (LDH) release in cell supernatants in all experimental conditions was not significantly elevated. The percentage of cell cytotoxicity was calculated by measuring the amount of LDH released from the damaged cells (1% triton x) into the supernatant. Data are shown as mean ± SEM of cell ratio (%). *** *p* < 0.001 vs. groups of corresponding genotypes (ANOVA, Bonferroni post-test). (**B**,**C**): IL-1β and caspase 1 levels in the supernatant of neutrophils. Values are expressed as mean ± SEM of target protein levels (pg/mL). *** *p* < 0.001, ** *p* < 0.01, * *p* < 0.05 vs. LPS-stimulated cells of corresponding genotype (ANOVA, Bonferroni post-test). (**D**,**G**) AnxA1 (37 kDa), pro-IL-1β (35 kDa), procaspase 1 (45 kDa) and cleaved caspase 1 (20–22 kDa) levels in the cell extracts under different experimental conditions. β-actin (42 kDa) was used as an endogenous control. (**E**,**F**,**H**,**I**): Immunoreactive bands were semi-quantified by densitometry and are expressed as arbitrary units (a.u.) of the ratio of AnxA1, pro-IL-1β, procaspase 1 and cleaved caspase 1/β-actin (representative image of three experiments performed). ** *p* < 0.01, * *p* < 0.05 vs. indicated cells of corresponding genotype; ^###^ *p* < 0.011, ^##^ *p* < 0.01 vs. same cell condition of different genotype (ANOVA, Bonferroni post-test). Data are representative of three independent experiments.

To analyze neutrophil viability, we carried out LDH assays on supernatants of WT and AnxA1^-/-^ neutrophils under different conditions: control (Opti-MEM), primed only (LPS), and primed and stimulated with nigericin or ATP, with or without Ac_2-26_ pre-treatment. All experimental conditions showed less than 30% of damaged neutrophils, as evidenced by LDH release (an indicator of cell cytotoxicity), compared to 1% triton x-treated cells (Figure 1A).

NLRP3 inflammasome activation by nigericin and ATP caused a significant increase in the release of IL-1β by WT neutrophils, an effect that was reduced by the addition of Ac_2-26_ (Figure 1B). These findings were corroborated by the immunoblot analysis of cell extracts, showing decreased levels of pro-IL-1β in Ac_2-26_-treated WT cells compared to non-treated cells (Figure 1D,F). Interestingly, nigericin administration was associated with high levels of AnxA1 in WT neutrophils (Figure 1D,E). AnxA1^-/-^ neutrophils were less responsive to nigericin stimulation compared to WT cells. Pre-treatment with Ac_2-26_ did not further reduce the lower level of IL-1β released by nigericin-stimulated AnxA1^-/-^ cells (Figure 1B). There was no difference in IL-1β release between ATP-stimulated and unstimulated LPS-primed AnxA1^-/-^ neutrophils (Figure 1B). Additionally, pro-IL-1β levels in AnxA1^-/-^ neutrophils presented an opposite pattern of expression of WT cells regarding Ac_2-26_ treatment (Figure 1D,F).

After NLRP3 activation by agonists, no significant change in caspase-1 release from WT and AnxA1^-/-^ neutrophils was detected, compared to primed-only cells (Figure 1C). However, immunoblot analysis indicated high expression of pro-caspase-1 in ATP-stimulated WT and AnxA1^-/-^ neutrophils, especially after pre-treatment with Ac_2-26_ (Figure 1G,H). Curiously, primed- and nigericin-AnxA1^-/-^ neutrophils showed higher levels of cleaved caspase-1 compared to WT neutrophils (Figure 1G,I). Ac_2-26_ addition produced increased levels of cleaved caspase-1 in WT neutrophils after ATP stimulation, while reduced levels of this isoform were detected in AnxA1^-/-^ cells after nigericin stimulation (Figure 1G,I). 

**Figure 2:**
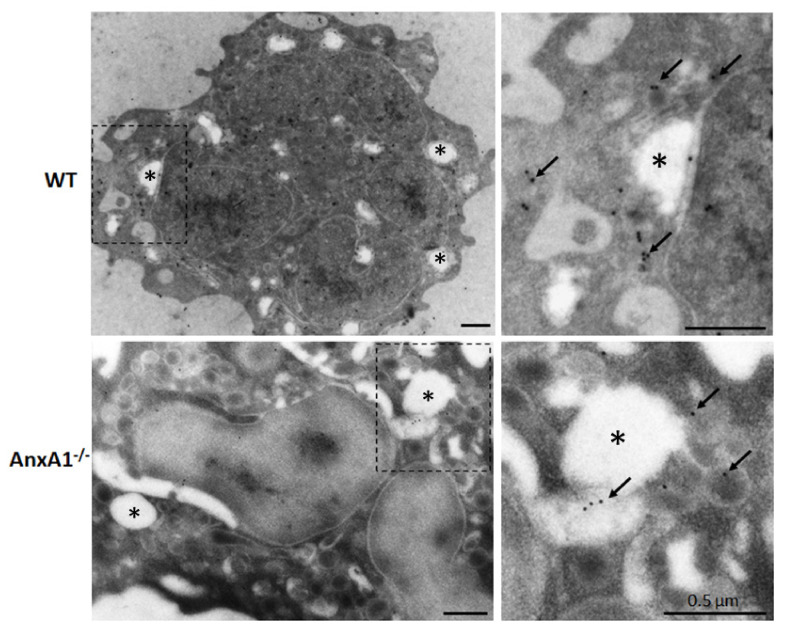
Expression of NLRP3 in WT and AnxA1^-/-^ nigericin-stimulated neutrophils. Immunogold labeling of NLRP3 (arrows) was detected in the cytoplasm of the neutrophils from both genotypes. These cells also contained cytoplasmic vacuoles (asterisks) that characterize their activation by nigericin. Images on the right side show details of the highlighted areas of the respective images on the left side. Representative images of three experiments performed.

Ultrastructural immunocytochemical analysis was carried out to confirm the expression of NLRP3 in neutrophils. Figure 2 shows that 15 nm gold particles targeted to NLRP3 are predominantly localized in the cytoplasm of nigericin-stimulated WT and AnxA1^-/-^ neutrophils.

### 3.2. NLRP3 Activation Alters Lipid Profile of WT and AnxA1^-/-^ Neutrophil Supernatants

**Figure 3:**
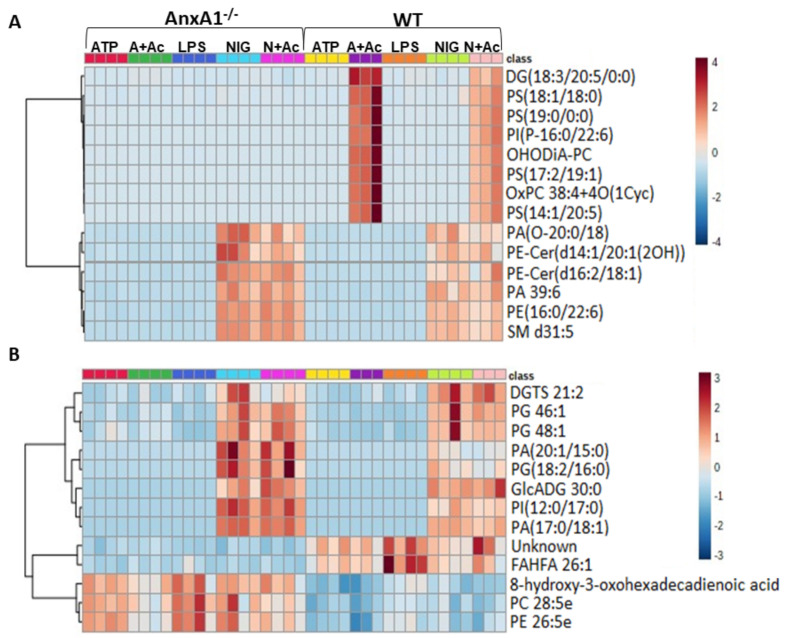
Lipidomic analysis of WT and AnxA1^-/-^ neutrophils supernatants. Heatmaps with dendrograms show the hierarchical clustering of potential lipid biomarkers. (**A**) Positive mode. (**B**) Negative mode. The right bar in (**A**,**B**) represents the blue-red code (−4 to 4 for positive and −3 to 3 for negative mode) of the lipid normalized peak areas. WT and AnxA1^-/-^ neutrophils were primed with lipopolysaccharide (LPS) and then stimulated with ATP, ATP with Ac_2-26_ pre-treatment (A + Ac), nigericin (NIG), and nigericin with Ac_2-26_ pre-treatment (N + Ac). Table S1 (Supplementary Material) shows the relevant chemical and statistical information for the discriminant lipids. Data are representative of one experiment performed.

To determine whether the lack of endogenous AnxA1 also alters the lipid profile of neutrophil under NLRP3 activation, a lipidomic analysis of cell supernatants was performed. In Figure 3, heatmaps and dendrograms highlight the normalized concentrations of different lipids in the supernatants of WT and AnxA1^-/-^ cells.

In the positive mode (Figure 3A), the majority of the potential lipid biomarkers were detected in supernatants from ATP- and nigericin-stimulated WT cells with Ac_2-26_ pre-treatment (A + Ac and N + Ac, respectively). These lipids were characterized by the glycerolipid diacylglycerol (DG)(18:3/20:5/0:0) and the glycerophospholipids phosphatidylserines (PS)(18:1/18:0), PS(19:0/0:0), PS(17:2/19:1), PS(14:1/20:5); phosphatidylinositol (PI)(P-16:0/22:6); and oxidized forms of phosphatidylcholines (PC) OHODiA-PC and OxPC 38:4 + 4O(1Cyc) (Figure 3A). In the positive mode, potential lipid biomarkers associated with nigericin-stimulated AnxA1^-/-^ and WT cells, with or without Ac_2-26_, were also detected. These nigericin-stimulated cells showed increased concentration of the glycerophospholipids phosphatidic acid (PA)(O-20:0/18:4), PA 39:6; phosphatidylethanolamine (PE)(16:0/22:6); and the sphingolipids sphingomyelin (SM) d31:5, and ceramide phosphoethanolamine (PE-Cer)(d14:1/20:1(2OH)), PE-Cer(d16:2/18:1) (Figure 3A). In the negative mode (Figure 3B), the glycerophospholipids phosphatidylglycerol (PG) 46:1, PG 48:1, PA(20:1/15:0), PG(18:2/16:0), PI(12:0/17:0) PA(17:0/18:1); and the glycerolipids diacylglyceryl-trimethylhomoserine (DGTS) 21:2 and glycosyldiradylglycerol (GlcADG) 30:0 were detected in supernatants from nigericin-stimulated AnxA1^-/-^ and WT cells, with or without Ac_2-26_ treatment.

WT and AnxA1^-/-^ cells also presented different lipid profiling in the negative mode analysis (Figure 3B). In WT supernatants, a high concentration of the fatty acid FAHFA 26:1 was detected, while AnxA1^-/-^ samples contained the fatty acid 8-hydroxy-3-oxohexadecadienoic acid, and the glycerophospholipids PC 28:5 and PE 26:5e. Table S1 shows the relevant chemical and statistical information for the discriminant lipids.

## 4. Discussion

Neutrophils are key players in the innate immune system [21] and are a potent source of the anti-inflammatory AnxA1 protein [22,23,24]. Our study evaluated the role of AnxA1 in NLRP3 inflammasome-derived IL-1β production by LPS-primed neutrophils following nigericin or ATP stimulation. Our study also provides a new perspective on the potential lipid biomarkers in neutrophils during NLRP3 activation through the mass spectrometry analysis of cell supernatants.

The inflammasome complex is a pivotal regulator for IL-1β processing, cleavage and releasing [2]. IL-1 isoforms and IL-18 are members of the IL-1-receptor/Toll-like receptor (IL-1R/TLR) superfamily [25]. After the recognition of damage-associated molecular patterns (DAMPs) and pathogen-associated molecular patterns (PAMPs) by TLRs the inflammasome can be oligomerize, driving to the IL-1β and IL-18 releasing [26]. Neutrophils express IL-1 receptors [27] and these receptors are increased after stimulation [28]. As we already know, during an inflammatory response neutrophils release IL-1β and other pro-inflammatory cytokines [29], but despite its response in inflammation, IL-1 isoform do not provoke a powerful neutrophil activation and basically their effect on neutrophils is to collaborate to the cell survival [28].

Our study demonstrated that stimulation with nigericin or ATP produced an increased release of IL-1β by WT neutrophils in a caspase-1-independent manner and without cell death, supporting unconventional protein secretion described previously [16,18,30,31]. The NLRP3/ASC/caspase-1 axis plays an important role in the IL-1 production by neutrophils, as cells null for NLRP3, ASC and caspase-1 did not release IL-1β after nigericin stimulation [16]. Besides caspase-1, studies have shown that neutrophil serine proteases, such as elastase and proteinase 3, are also involved in the processing of pro-IL-1β [30,32,33]. In fact, more than one pathway seems available for processed IL-1β to exit the cell, including exocytosis of the secretory lysosomes, shedding of plasma membrane microvesicles, direct release via transporters, or multivesicular bodies containing exosomes [34].

Canonical inflammasome pathway is based on the cleavage of pro-IL1β by caspase 1, as well as the gasdermin-D (GSDMD) to a 31 kDa N-GSDMD molecule. Thus, N-GSDMD oligomerizes in the cell plasma membranes to increases the membrane permeability and release of cleaved IL-1β, a mechanism that drives to pyroptosis [35]. As it has been already elucidated, neutrophils release IL-1β without pyroptosis process in a canonical NLRP3 inflammasome activation [18,36,37]. Recently it was demonstrated that although N-GSDMD is required for IL-1β secretion in NLRP3 inflammasome activation, N-GSDMD is not part of this system in human and murine neutrophils [38]. Basically, N-GSDMD is associated with azurophilic granules and LC3+ autophagosomes present in the neutrophil cytosol, which leads to a secondary cleavage of GSDMD indicating that IL-1β is secreted by neutrophils by an autophagy-dependent mechanism [38].

Our study also showed that the lack of endogenous AnxA1 is associated with higher levels of cleaved caspase-1 after nigericin and ATP stimulation compared to WT neutrophils, despite that no increase in IL-1β release was detected. In neutrophils, endogenous AnxA1 promotes specific granule self-aggregation, fusion of granules, and their coaggregation with plasma membrane vesicles in a calcium-dependent manner [39,40]. AnxA1^-/-^ neutrophils are also more susceptible to activation by different inflammatory stimuli, such as platelet-activating factor, fMLP, and phorbol myristate acetate, with increased levels of CD11b in comparison to WT cells [17]. Indeed, AnxA1^-/-^ mice show a greater influx of neutrophils and a consequent exacerbation of the inflammatory response in different experimental models of inflammation [11,17,41,42,43].

Unlike neutrophils, macrophages isolated from AnxA1^-/-^ C57BL/6 mice have higher levels of cytoplasmic NLRP3 and, when stimulated by nigericin, release increased levels of IL-1β compared to WT cells. The points of AnxA1 and NLRP3 co-localization were identified in WT nigericin- or MSU crystal-stimulated macrophages, as detected by immunofluorescence and ultrastructural analysis [13,14]. Although the biology of NLRP3 has been widely studied in macrophages and dendritic cells, studies have shown that neutrophils are the main source of the NLRP3-dependent IL-1β production in *Staphylococcus aureus* [44] and *Streptococcus pneumoniae* mouse infections [36]. It is known that the immune signaling pathways usually exhibit specific effects applicable to each cell type, including the inflammasome signaling. For example, studies using different agonists for NLRP3 have shown that neutrophils respond differently to macrophages with regards to NLRP3 activation [45]. In neutrophils, NLRP3 shows less responsiveness to the agonists alum, silica, and MSU crystals compared to macrophages, in which these compounds trigger NLRP3 activation by phagolysosomal disruption [45]. Furthermore, murine neutrophils are poorly responsive to the lysosomotropic peptide Leu-Leu-OMe, suggesting that lysosomal disruption is not the usual mechanism for NLRP3 activation in neutrophils. On the other hand, robust and NLRP3-dependent responses were observed to the agonists nigericin, ATP, and R837, which mimic the presence of infection or tissue damage [45]. Considering that the signaling pathways for NLRP3 activation in macrophages are not always conserved in neutrophils, our findings demonstrate that endogenous AnxA1 may also have a strong influence on cell identity, particularly in innate immune signaling pathways. Thus, we hypothesize that the lack of endogenous AnxA1 in neutrophils causes a less efficient activation of NLRP3 due to an imbalance in the activation of caspase 1 and the membrane fusion system, resulting in a less effective release of IL-1β.

In the current study, the administration of AnxA1-derived peptide Ac_2-26_ showed a regulatory effect on NLRP3 activation through the reduction of the IL-1β release by nigericin and ATP-stimulated WT neutrophils. This is consistent with a previous report using monosodium urate (MSU) crystal-induced gout in mice which showed that pre- or post-treatment with Ac_2-26_ reduces the migration of neutrophils into the synovial cavity, as well as the production of IL-1β in synovial tissue [11]. On the other hand, blocking Fpr or AnxA1 protein caused an increase in the influx of neutrophils into the synovial cavity. Considering that in the gout model the NLRP3 inflammasome leads to the production of IL-1β and that neutrophils represent the main source of this cytokine [46,47], our findings indicate that the regulatory effect of Ac_2-26_ on NLRP3 activation could be via Fpr. Ac_2-26_ can bind to Fpr1 and Fpr2 through which it regulates the activation of neutrophils [48] and, the relationship of Fpr1 in the activation of the NLRP3 inflammasome was recently demonstrated [49].

Although similar NLRP3 levels were detected in the cytoplasm of AnxA1^-/-^ and WT neutrophils, as shown in the ultrastructural analyzes, AnxA1^-/-^ neutrophils were less responsive to nigericin and ATP stimulation compared to WT cells and released low levels of IL-1β. The administration of AnxA1-derived peptide Ac_2-26_ neither significantly reduced IL-1β, as seen in stimulated WT cells, nor increased (as compensatory mechanism), indicating an important role of the endogenous form of AnxA1 in the activation of the NLRP3 platform.

Another aspect explored in this study was the lipid profiling of WT and AnxA1^-/-^ neutrophil supernatants under NLRP3 activation using mass spectrometry analysis. Lipids are crucial in many biological functions, such as structural components of lipid bilayers, energy storage, and metabolism, signaling pathways, and lipidomics represents a powerful tool for the study of lipid biomarkers in several experimental approaches and diseases [50]. In our study, the most of the potential lipid biomarkers detected in neutrophil supernatants were glycerophospholipids (PS, PC, PE, PA, PG, and PI), key components of the lipid bilayer of cells [50], suggesting that microparticles are released by neutrophils under LPS priming and NLRP3 inflammasome activation. Stimulated neutrophils produce microparticles with rapid and anti-inflammatory properties, in vitro and in vivo, reliant on their expression of AnxA1 [51,52,53].

We provide evidence that nigericin stimulation produces high amounts of sphingolipids PE-Cer(d14:1/20:1(2OH)), PE-Cer(d16:2/18:1) and SM d31:5 in the supernatants of WT and AnxA1^-/-^ mice. Sphingolipids contain common core structures which are converted into ceramides and other classes of sphingolipids [50,54]. It is known that NLRP3 activation promotes the production of lipid mediators, for example ceramides, that trigger signaling pathways during inflammation [55] and also in cell death [56]. In addition, IL-1β induces ceramide synthesis in human neutrophils; and inhibition of IL-1β binding (by pre-treatment with IL-1RA) or ceramide synthesis attenuates NETosis [57].

Nigericin stimulation also increased the concentration of the glycerophospholipids PA (phosphatidic acid) and PC (phosphatidylglycerol) in neutrophil supernatants. Like sphingolipids, changes in the glycerophospholipid metabolic network can alter cellular functions, such as membrane fusion, fission, and vesicle transport, and influence membrane potential and ion transport [50]. Nigericin induced K^+^ efflux and Ca^2+^ influx signaling which culminated in the NLRP3 activation and IL-1β and IL-18 release, ROS production, and endoplasmic reticulum stress [30,58,59].

The administration of AnxA1-derived peptide Ac_2-26_ altered the lipid profiling of supernatants from WT nigericin- and ATP-stimulated neutrophils, revealing increased concentration of PS(18:1/18:0); PS(19:0/0:0); PS(17:2/19:1) and PS(14:1/20:5) and two PC in their oxidized forms, OHODiA-PC and OxPC 38:4 + 4O(1Cyc). AnxA1 is a 37-kDa calcium-dependent phospholipid-binding protein that has a specific interaction with PS and PC [60]. Upon activation with tumor necrosis factor, neutrophils rapidly release microvesicles that express AnxA1 and/or PS exposed on their outer membrane leaflet which attenuate macrophage activation in response to LPS and interferon gamma (IFN-γ) [61]. During LPS-induced pleurisy in mice, treatment with Ac_2-26_ promoted the resolution of neutrophilic inflammation in the pleural cavity, an effect associated with the induction of neutrophil apoptosis [62]. The process of apoptosis also increased the content of biologically active oxidized phospholipids [63]. Upon oxidation of phosphatidylcholine, the PC head group becomes exposed and available for recognition by immune receptors [64]. Despite driving acute inflammation, neutrophil-derived microvesicles are known to promote tissue protection and repair by affecting the function and phenotype of target cells [51,53]. Therefore, we speculate that Ac_2-26_ regulates ATP- and nigericin-stimulation of NLRP3 by triggering the production of anti-inflammatory microvesicles and the proapoptosis pathway in neutrophils and, consequently, the resolution of inflammation.

As a final observation, supernatants from WT and AnxA1^-/-^ neutrophils presented different lipid profiling, independent of NLRP3 activation and Ac_2-26_ treatment. In WT samples, a high concentration of the fatty acid FAHFA 26:1 was detected, while the 8-hydroxy-3-oxohexadecadienoic acid and two glycerophospholipids (PC 28:5 and PE 26:5e) were detected in AnxA1^-/-^ samples. Given that AnxA1 is a phospholipid-binding protein that participates in the granule aggregation/fusion with the plasma membrane in neutrophils [39,40], it is reasonable to suppose that the lack of it causes a lipid bilayer alteration compared to WT.

## 5. Conclusions

In summary, the results show that AnxA1 may play different roles in the NLRP3 inflammasome signaling based on its intra or extracellular localization and cellular type. In neutrophils, endogenous AnxA1 is essential to activate the NLRP3 machinery and release IL-1β, but once in the extracellular microenvironment, it acts to control the release of this cytokine and the consequent inflammatory response.

## Data Availability

Data available on request due to restrictions.

## Supplementary Materials

The following are available online at https://www.mdpi.com/2073-4409/10/1/121/s1, Table S1: Statistical and chemical data of differential features.
