# Supplementary material for: Role of Annexin A1 in NLRP3 Inflammasome Activation in Murine Neutrophils
Source: Cells. 2021 Jan 11;10(1):121. doi: 10.3390/cells10010121 (PMC7827236; doi:10.3390/cells10010121)
Supplement: Supplementary file 1 [file cells-10-00121-s001.pdf]

Table S1: Statistical and chemical data of differential features

Name	Peak (rt_m/z or n)	ESI[a]	p-value[b]	FDR	Molecular Formula	Mass Error
PS(19:0)	0.47_539.3224n	+	8.82E-13	5.75E-12	C25H50NO9P	0.20
PI(P-16:0/22:6)	0.47_930.5470m/z	+	3.89E-12	1.60E-11	C47H79O12P	0.40
OHODiA-PC	0.50_692.4100m/z	+	4.63E-14	5.50E-13	C34H62NO11P	-4.82
PS(17:2/19:1)	0.48_824.4872m/z	+	5.88E-13	4.16E-12	C42H76NO10P	4.32
OxPC 38:4+4O(1Cyc)	0.48_912.5366m/z	+	9.33E-13	5.93E-12	C46H84NO12P	0.42
PS(14:1/20:5)	0.45_751.4436n	+	1.90E-12	9.38E-12	C40H66NO10P	1.51
PS(18:1/18:0)	0.48_828.5141m/z	+	2.99E-14	3.87E-13	C42H80NO10P	-1.34
DG(18:3/20:5/0:0)	0.79_675.4359m/z	+	1.03E-21	1.12E-19	C41H64O5	-4.13
PA(O-20:0/18:4)	3.80_675.5109m/z	+	2.54E-13	2.07E-12	C41H75O7P	-0.39
PE-Cer(d14:1/20:1(2OH))	3.82_639.4884m/z	+	1.48E-09	4.21E-09	C36H71N2O7P	3.54
PA 39:6	3.10_773.4482m/z	+	8.34E-14	9.01E-13	C42H71O8P	-4.91
PE-Cer(d16:2/18:1)	3.80_657.4981m/z	+	1.67E-11	6.13E-11	C36H69N2O6P	2.28
PE(16:0/22:6)	2.93_728.5032m/z	+	5.47E-19	1.46E-17	C43H74NO8P	2.45
SM d31:5	3.80_675.4461m/z	+	6.85E-20	2.33E-18	C36H65N2O6P	-1.81
FAHFA 26:1	5.44_459.3264m/z	-	1.54E-09	1.54E-09	C26H48O4	4.06
8-hydroxy-3-oxohexadecadienoic acid	0.86_263.1661m/z	-	2.99E-08	2.99E-08	C16H26O4	2.85
PC 28:5e	12.33_634.4246m/z	-	8.95E-08	8.95E-08	C36H64NO7P	0.64
PE 26:5e	1.96_564.3475m/z	-	1.62E-07	1.62E-07	C31H54NO7P	2.57
DGTS 21:2	16.41_534.3790m/z	-	6.00E-08	6.00E-08	C31H55NO7	-1.78
PG 46:1	15.30_961.7078m/z	-	1.67E-09	1.67E-09	C52H101O10P	-4.01
PG 48:1	15.75_989.7448m/z	-	3.00E-08	3.00E-08	C54H105O10P	2.16
PA(20:1/15:0)	4.38_723.4724m/z	-	4.59E-09	4.59E-09	C38H73O8P	-1.85
PG(18:2/16:0)	4.38_781.4776m/z	-	4.07E-08	4.07E-08	C40H75O10P	-2.07
PI(12:0/17:0)	3.80_768.4817n	-	2.16E-15	2.16E-15	C38H73O13P	3.61
PA(17:0/18:1)	3.80_723.4714m/z	-	8.45E-21	8.45E-21	C38H73O8P	-3.40
GlcADG 30:0	3.66_737.4829m/z	-	1.58E-13	1.58E-13	C39H72O11	1.01

a) Electrospray Ionization, positive or negative mode. b) FDR adjusted
